# Development of an Intranasal In Situ System for Ribavirin Delivery: In Vitro and In Vivo Evaluation

**DOI:** 10.3390/pharmaceutics16091125

**Published:** 2024-08-26

**Authors:** Iosif B. Mikhel, Elena O. Bakhrushina, Danila A. Petrusevich, Andrey A. Nedorubov, Svetlana A. Appolonova, Natalia E. Moskaleva, Natalia B. Demina, Svetlana I. Kosenkova, Mikhail A. Parshenkov, Ivan I. Krasnyuk, Ivan I. Krasnyuk

**Affiliations:** 1 A.P. Nelyubin Institute of Pharmacy, I.M. Sechenov First Moscow State Medical University (Sechenov University), Moscow 119048, Russia; bakhrushina_e_o@staff.sechenov.ru (E.O.B.); petrusevichdan@yandex.ru (D.A.P.); demina_n_b@staff.sechenov.ru (N.B.D.); kosenkova_s_i@staff.sechenov.ru (S.I.K.); misjakj@gmail.com (M.A.P.); krasnyuk_i_i_1@staff.sechenov.ru (I.I.K.J.); krasnyuk_i_i@staff.sechenov.ru (I.I.K.); 2 Institute of Translational Medicine and Biotechnology, I.M. Sechenov First Moscow State Medical University (Sechenov University), Moscow 119048, Russia; nedorubov_a_a@staff.sechenov.ru; 3 Centre of Biopharmaceutical Analysis and Metabolomics, I.M. Sechenov First Moscow State Medical University (Sechenov University), Moscow 119048, Russia; appolonova_s_a@staff.sechenov.ru (S.A.A.); moskaleva_n_e@staff.sechenov.ru (N.E.M.)

**Keywords:** intranasal administration, in situ systems, ribavirin, nose-to-brain mechanism, cancer, anticancer therapy, in vitro tests, in vivo tests, Quality by Design approach

## Abstract

Recently, ribavirin has demonstrated effectiveness in treating glioblastoma through intranasal administration utilizing the nose-to-brain delivery route. Enhancing ribavirin’s bioavailability can be achieved by utilizing intranasal stimuli-responsive systems that create a gel on the nasal mucosa. The research examined thermosensitive, pH-sensitive, and ion-selective polymers in various combinations and concentrations, chosen in line with the current Quality by Design (QbD) approach in pharmaceutical development. Following a thorough assessment of key parameters, the optimal composition of gellan gum at 0.5%, Poloxamer 124 at 2%, and purified water with ribavirin concentration at 100 mg/mL was formulated and subjected to in vivo testing. Through experiments on male rats, the nose-to-brain penetration mechanism of the active pharmaceutical ingredient (API) was elucidated, showcasing drug accumulation in the olfactory bulbs and brain.

## 1. Introduction

According to statistics from the World Health Organization (WHO) in 2022, there were more than 20 million new cases of cancer and 9.7 million deaths. According to current predictions, by 2050, the incidence of cancer could increase by 77% and deaths from cancer could double [1]. With such prospects, the development of drugs that can prevent or slow down the rapid increase in the incidence of deadly pathologies becomes the main agenda. Statistically few but extremely aggressive types of neoplasms include brain tumors (gliomas, glioblastomas, astrocytomas, meningiomas, etc.), which in the absence of proper therapy or surgical intervention lead to life-threatening and, in 7% of cases, fatal outcomes for patients.

The significance of accelerating the pace of drug development is evident, yet the potential for serious, life-threatening adverse drug events (ADEs) to impede or halt the research process cannot be overlooked. It is, however, unfeasible to envisage a new drug development cycle without comprehensive preclinical and clinical studies of efficacy and safety. To a certain extent, the drug development process can be facilitated by the utilization of existing active pharmaceutical ingredients (APIs) for new off-label indications. The discovery of a novel delivery mechanism, route of administration, or modification of the structure of an existing API enables the development of a drug with a novel spectrum of action.

In light of the ever-increasing need for effective antiviral agents, ribavirin can currently be used in the pharmacotherapy of chronic hepatitis E, Lassa fever, fever with renal syndrome caused by the Hantaan virus, respiratory syncytial virus, COVID-19 infection, and possibly gastroenteritis caused by astroviruses. The results of recent studies indicate that ribavirin may be an effective treatment for certain histological types of nasopharyngeal carcinoma, breast cancer, lung cancer, colorectal cancer, hepatocellular carcinoma, HPV-associated malignancies, ovarian cancer, a number of hemoblastoses, and soft tissue sarcoma [2,3,4,5,6,7,8,9,10,11,12,13,14,15,16,17,18,19].

Due to the aggressive nature of brain tumors, ribavirin could be a suitable agent for direct targeting of the tumor site. While complete degradation is unlikely, it may be effective for reducing the size of the tumor prior to surgical intervention. At present, clinical trials of ribavirin as an antitumor agent are being conducted, either as monotherapy or in combination with other agents [11,20,21].

In accordance with the prevailing biopharmaceutical classification system, ribavirin is classified as a class III API (high solubility, low permeability). This suggests that a promising strategy for modification may be to enhance the degree of its penetration through histohematic barriers, prolongation of action by increasing mucoadhesion, and increased affinity to lipophilic structures of the body using appropriate polymeric carriers or a novel method of delivery of the studied drug.

In contemporary therapeutic practice, ribavirin is administered orally or topically. When administered orally, the bioavailability of ribavirin is approximately 45%. The biological fluids of the gastrointestinal tract have a significant impact on the bioavailability of drugs, with only a small proportion of the administered dose entering the systemic bloodstream and exerting its pharmacological effect. Nevertheless, the liver is the organ where ribavirin undergoes biotransformation to its active metabolite—phosphate [22,23].

### 1.1. Specifics of Intranasal Administration

The administration of drugs via the nasal route allows for the achievement of optimal therapeutic outcomes due to the presence of extensive blood vessels in the nasal cavity, a large injection site (~160 cm^2^), and the rapidity of pharmacological action. Among other advantages, intranasal delivery represents a more appealing alternative for patients undergoing therapy compared to invasive routes of drug administration. Another noteworthy benefit of intranasal delivery is the capacity to transport APIs from the nasal cavity directly to the brain, a process known as nose-to-brain delivery. This delivery mechanism is possible due to the penetration of APIs through cranial nerves innervating the nasal cavity. The study by Colombo et al. was one of the first to describe studies of ribavirin absorption in the nasal cavity and its distribution in the brain. Ribavirin was administered both intranasally and intravenously to compare bioavailability. In the first in vivo experiment, an aqueous solution of ribavirin was administered intranasally to Sprague–Dawley rats (10 mg/kg). The study was conducted in different hemispheres and brain regions (cerebellum, olfactory bulb, cerebral cortex, basal ganglia, and hippocampus) and biological fluids (cerebrospinal fluid and plasma). Ribavirin was quantified using mass spectrometry. By instrumental analysis, API was detected in all compartments at each time point, with the highest concentration in the olfactory bulb and a decrease in the rostro–caudal direction. Two subsequent in vivo experiments compared the nasal route of administration (ribavirin solution) with intravenous and nasal administration of ribavirin solution with ribavirin powder for intranasal administration (10 mg/kg) [24]. It was found that 20 min after administration, the concentration of ribavirin in the olfactory bulb was similar after intravenous or nasal administration of ribavirin solution, whereas the powder resulted in significantly higher levels. Although the intravenous route of administration is the most effective, the basal ganglia and hippocampus were shown to be clearly superior to the intranasal administration of API when analyzing the basal ganglia and hippocampus [24].

Despite the numerous advantages of intranasal administration, there are inherent limitations that make it challenging to deliver drugs through the nasal cavity [25]. The constant renewal of the mucosa by cilia movement and mucus secretion by secretory cells (mucociliary clearance) provides a rapid removal of drugs and other foreign bodies from the surface of the nasal cavity. The incomplete and transient retention of the drug on the mucosal surface precludes the complete release of the API from the matrix. This frequently results in an overestimation of the dose of the API. Furthermore, the intricate geometry of the nasal cavity necessitates the development of specialized nebulizer systems to direct the drug to the olfactory bulb for subsequent transport of the API to the brain [26,27].

These disadvantages constrain the use of intranasal drug delivery via the nose-to-brain route. However, there are mechanisms that can compensate for the shortcomings of nasal dosage forms. Stimulus-sensitive (in situ) systems represent a solution to the problems of intranasal dosage forms. In situ delivery systems perform sol–gel transition when exposed to different gelation stimuli, including temperature, ionic composition, pH, moisture, and solvent diffusion into the surrounding soft tissue. Depending on the gelation stimulus, in situ smart polymers are divided into groups: thermoreversible, ion-triggered, pH-sensitive, phase-inversion, photosensitive, and moisture-activated [28,29,30].

### 1.2. Selection of an Optimal Gelation Stimulus

The gelation triggers that enable the phase transition of stimuli-sensitive systems can be divided into two main categories: physical and chemical. In most cases, smart polymers with a physical crosslinking mechanism are preferred for in situ gelation in the nasal cavity due to their rapid gelation and safety profile. This gelation mechanism employs temperature, pH, and ions as the triggering stimuli [31,32,33]. The use of smart polymers with other gelation stimuli increases the probability of adverse events and toxic effects at the site of drug application, namely at the nasal mucosa [31]. For instance, the gelation of photosensitive polymers is accompanied by the release of heat due to an exothermic reaction, which can have a deleterious effect on the mucosa. One limitation of phase-inversion polymers is the presence of a solvent in the system, which must diffuse into the surrounding soft tissues to form a gel. It is not uncommon for solvents utilized in such systems to be organic in nature (e.g., N-methylpyrrolidone, DMSO, and triacetin) [34]. Their introduction into nasal cavity tissue can result in alterations to the natural mucociliary clearance process and the olfactory function of the nose. While moisture-activated polymers demonstrate excellent gel-forming capabilities, their utility in the fabrication of intranasal in situ systems remains debatable. Since moisture-activated systems are represented by powder compositions, the instinctive defense mechanisms against foreign bodies (sneezing) may be triggered when they are atomized in the nasal cavity, which will lead to the removal of a significant part of the drug from the mucosal surface and, consequently, an irritating effect. pH-sensitive polymers—e.g., chitosan salts (and gazetteer references) as a mono-stimulant did not allow for gelation in the physiologic range, and carbopols used by some authors have acidic pH media and can cause cyto- and cyliotoxicity when applied [35,36,37].

The results of the literature review indicated that thermoreversible and ion-selective polymers are the most suitable for the development of intranasal in situ systems. These polymers exhibit phase transition ability under natural conditions of the nasal cavity. These types of smart polymers are capable of rapidly forming gels in the nasal cavity and exhibit acceptable physicochemical properties for performance.

Ion-selective polymers have a relatively simple gelation mechanism. The best-known ion-selective polymers available on the market are gums (gellan gum, xanthan gum, guar gum) and pectins with different degrees of esterification. The three-dimensional networks formed by ion-selective polymers during gelation are based on so-called “egg box” structures. Linear polysaccharides are bound by calcium cations or other cations in chains that, branching and distributed according to the principle of maximum conservation of free energy, form a network resembling a honeycomb of irregular shape [38].

According to the literature, thermosensitive polymers are inherently amphiphilic polymers with hydrophilic and hydrophobic groups that are potentially capable of forming micelles. A change in the solubility of the polymer in water is required for the phase transition to occur. Thermosensitive polymers are typically used at high concentrations in targeted delivery systems due to the need to overcome the critical micelle formation concentration for a temperature-dependent phase transition. To achieve the temperature-dependent sol–gel transition, most researchers use a commercially available polymer called Poloxamer 407. Directed synthesis of thermosensitive excipients has also been developed in recent years, most commonly using copolymers of poly(lactic-co-glycolic acid)—PLGA, as the base. Usually, in situ gelation of thermosensitive polymers is performed in two steps—upon reaching the critical temperature, micelles are formed from linear polymers, and after passing the temperature limit of gelation—micelles are assembled into a three-dimensional network. A similar mechanism is characteristic of Poloxamer 407 [39,40,41].

In addition to the basic polymers, most systems require supporting excipients to ensure optimal performance of the final delivery system. Polyvinyl alcohol (PVA) and polyethylene glycol (PEG) are the most commonly used excipients. These excipients are used to create their macroporous structure and to form the micellar structure of the sol. In addition to these effects, mucoadhesives (chitosan, Poloxamer 124, hydroxypropylmethylcellulose) are necessary for a high-quality delivery system. Due to their high affinity for mucin on the mucosal surface, these substances bind to mucin and form strong bonds, allowing the drug to be retained and released for longer periods of time [42,43,44].

A considerable number of studies have been conducted to describe the development of stimulus-sensitive systems for the delivery of various APIs. The long drug development process necessitates the utilization of novel tools that can accelerate the research and development phase. To achieve this goal, new experimental design engineering techniques are being investigated. One of the most effective and extensively studied methods for enhancing the efficiency of the development process is the design of a study in accordance with the principles of Quality by Design (QbD) [45,46,47,48].

The fundamental principle underlying the QbD concept is the sequential characterization of the product to be developed. Firstly, quality target product parameters (QTPP) are identified, which include product characteristics that guarantee the efficacy and safety of the final medicinal product. Based on the QTTP, the physical, chemical, and biological characteristics of the final product—critical quality attributes (CQAs)—are distinguished. The critical material attributes (CMAs) and critical process parameters (CPPs) exert a significant influence on the results of the critical quality attributes (CQAs), which are classified as high-risk factors [45,46,47,48].

The aim of this study was to develop a stimuli-responsive ribavirin delivery system capable of penetrating the brain via a nose-to-brain mechanism, potentially with potential promise for use in brain tumor therapy.

## 2. Materials and Methods

### 2.1. Equipment

For the experiment, we used a magnetic stirrer (IKA C-mag Hs7 digital, Staufen im Breisgau, Germany), a stirrer with dynamic viscosity control (IKA STARVISC 200-2.5 control, Staufen im Breisgau, Germany), a high-pressure liquid chromatograph Agilent 1290 Infinity II (Agilent Technologies^®^, Santa Clara, CA, USA) with a tandem quadrupole mass spectrometry detector Agilent 6470 Triple Quad LC/MS (Agilent Technologies^®^, USA), a pH meter (Starter 2100 pH Bench pH Meter ST2100-F, Shanghai, China), a coaxial cylinder viscometer (Lamy Rheology RM 200, Paris, France), and an in vitro model of the nasal cavity (developed at the Institute of Pharmacy named after A.P.Nelyubin, Sechenov University, Moscow, Russia).

### 2.2. Excipients and Chemicals

The thermoreversible in situ polymer used in this study was Poloxamer 407 (P407) (Kolliphor^®^ P407, BASF, Ludwigshafen, Germany); the ion-selective in situ polymers were gellan gum (GG) (Molecularmeal, Moscow, Russia), xanthan gum (XG) (Vanzan NFC, Vanderbilt Minerals, Gouverneur, NY, USA), guar gum (GuG) (Molecularmeal, Moscow, Russia), and pectin (Pec) (Molecularmeal, Moscow, Russia); the mucoadhesive polymers were hydroxypropyl methylcellulose (HPMC) (Ashland, Wilmington, DE, USA), Poloxamer 124 (P124) (Kolisolv^®^ P124 Geismar, BASF, Ludwigshafen, Germany), polyvinyl alcohol (PVA) (Prime Chemicals Group, Moscow, Russia), chitosan 200 (Ch) (SigmaChem. Co., Burlington, MA, USA), and Carbopol^®^971 (Carb) (Lubrizol, Wickliffe, OH, USA); and the excipients were phosphate buffer (PBS), polyethylene glycol (m.w.—1500) (PEG-1500) (Prime Chemicals Group, Moscow, Russia), and Poloxamer 188 (P188) (Kolliphor^®^ P 188 Geismar, BASF, Ludwigshafen, Germany). Other chemicals used were sodium chloride—NaCl (HiMedia Laboratories, Maharashtra, India); potassium chloride—KCl (HiMedia Laboratories, Maharashtra, India); calcium chloride—CaCl_2_ (Sisco Research Laboratories, Mumbai, India); sodium hydroxide—NaOH (HiMedia Laboratories, Maharashtra, India); formic acid—HCOOH (Chimmed, Moscow, Russia); heptafluorobutyric acid—C3F7COOH (Chimmed, Moscow, Russia); ammonium formate—HCOONH4 (Chimmed, Moscow, Russia); and acetonitrile—CH3CN (Sigma-Aldrich, Burlington, MA, USA). The purity of chemicals was “Pro Analysi”—pure for analytical studies (99%). Ribavirin was obtained from Chimmed (Moscow, Russia)—99% pure (Pro Analysi).

### 2.3. Design of Experiment

This study employed a QbD methodology to design the experiments. According to this concept, the initial step was to define the quality target product profile (QTPP) of the in situ system being developed for intranasal delivery of ribavirin (Table 1).

Subsequently, the proposed and justified QTPP was utilized to identify critical quality attributes (CQA), critical material attributes (CMA), and critical process parameters (CPP). The aforementioned list is presented in Table 2.

In order to ascertain the impact of process parameters on critical quality indicators, the compositions were prepared with varying process conditions. These included stirring time (10 min and 60 min), stirring speed (50 rpm and 500 rpm), and temperature during manufacturing (up to 25 °C and above 70 °C). The subsequent step was to ascertain the impact of the formulation components on the critical parameters. The following were proposed as critical parameters of the formulation: concentration of the gelling agent—minimum and maximum concentrations (*w*/*v*%) were used as boundary values; additional ingredients, including mucoadhesive polymers and excipients that enhance the technological characteristics of the final drug—minimum and maximum concentrations (*w*/*v*%) were used; concentration of API—ribavirin was administered at a concentration of 100 mg/mL.

**Figure 1:**
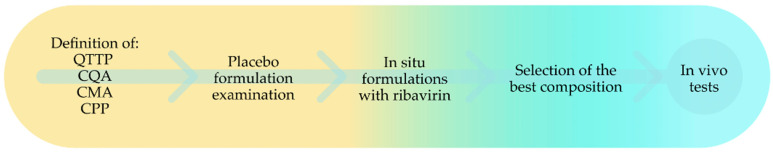
Sequence of work according to the QbD concept.

The obtained formulations were subjected to testing for critical quality parameters, and the results were analyzed using MiniTab 17.0 software. The program defined the degree of influence of factors as high, medium, or low. A brief schematic of the development process is shown in Figure 1.

### 2.4. Methods

#### 2.4.1. In Vitro Studies

##### Determination of In Situ Gelation Ability

Each composition (1 mL) was subjected to conditions analogous to those of the nasal cavity, contingent on the in situ gelation stimulus. All measurements were carried out at a temperature of 32 °C and in the medium simulated nasal cavity secretion (2:1) containing an equivalent physiologic concentration of Na^+^, K^+^, and Ca^2+^ ions (8.77 mg/mL NaCl, 2.98 mg/mL KCl and 0.59 mg/mL CaCl_2_) with a pH of 6.5 ± 0.5. The thermoreversible compositions’ in situ sol–gel transition ability was evaluated by heating them in a water bath and recording the sol–gel phase transition temperature (2.4.4. Determination of gelation temperature) [49]. Gel formation was recorded using an IKA STARVISC 200-2.5 control (Germany) stirrer with dynamic viscosity control. Gel formation was detected by a significant increase in dynamic viscosity and also visually.

##### pH Measurement

Polymer compositions were subjected to potentiometric testing following fabrication and cross-linking. This was conducted using a pH meter (Starter 2100 pH Bench pH Meter ST2100-F, Ohaus Instruments Co.,Ltd., Shanghai, China). Formulations with a pH below 5.50 or above 8.50, which are outside the physiological pH range of the nasal cavity, were excluded from the study because they could cause ciliotoxic effects on the mucosa.

##### Viscosity Measurement

The tests were performed at 20 °C over a range of shear rates from 0 to 300 s^−1^ for 60 s. To obtain the average results, three measurements were taken for each sample after half an hour of relaxation. The stability of the index was determined by calculating the plastic viscosity using the Casson model [50,51].

##### Gelation Temperature

The phase transition temperature of thermoreversible compositions was investigated using an ODA-MH13 ultrasonic bath (Shen Zhen Derui Ultrasonic Equipment Co., Ltd, Shenzhen, China) heated to 50 °C. The temperature at which the entire volume of the analyzed sample (20 mL) underwent a phase transition from a sol to a gel was recorded using a thermal probe [49].

##### Gelation pH

The pH of the phase transition was determined using a pH meter (Starter 2100 pH Bench pH Meter ST2100-F, China). Subsequently, the pH of the sol was measured, and an alkaline agent was gradually added to the composition, which was then stirred on a magnetic stirrer (IKA C-mag Hs7 digital, Staufen im Breisgau, Germany). The pH at which the entire volume of the analyzed sample (20 mL) was transferred from the sol to the gel was recorded.

##### Determination of the Completeness of Retention on the Mucosal Surface In Vitro

The research was conducted using an in vitro model of the nasal cavity. The in vitro model of the nasal cavity was obtained by 3D printing on a ZAV-big printer using glycol-modified polyethylene coated with ACRYL-ART Titanium White acrylic paint (Russia) and DECOLA gloss acrylic paint (Russia) for better adhesion and durability. The model was made 1:1 based on the results of the study by Liu et al. on 60 healthy volunteers and the structure of their nasal cavity using computed tomography [52]. For ease of use, the model was placed on a special stand that allowed it to maintain a physiological tilt of 25° during use. The developed model was irrigated before each experiment with a solution reproducing the mineral composition of nasal fluid: 8.77 mg/mL NaCl, 2.98 mg/mL KCl, 0.59 CaCl_2_ mg/mL (pH = 6 ± 0.5), and porcine gastric mucin type II (Sigma-Aldrich, Burlington, MA, USA) 4% (*w*/*v*%) [36]. After application of the irrigation solution, the model was covered with a film also pretreated with irrigation solution (0.1 mL). A polymer collector was attached to the nasopharyngeal outlet to collect the sample leaving the model. The study samples were injected into the model through a spray system at a 45° angle. The samples were introduced using a spray syringe (Yelets MPK, Lipetsk, Russia). The model was then placed in a Binder FD 115 climatic chamber (Binder, Bohemia, NY, USA) and incubated for 5 min at 37 ± 0.5 °C. After remaining in the thermostat, the receiver containing the sample evacuated during the experiment was removed and the outflow volume was recorded [36].

##### Spray Torch Measurement

A spray syringe (Yelets MPK, Lipetsk, Russia) was used to apply 1 mL of the composition colored with water-soluble dye (Brando, Russia) onto non-woven (white spunbond, PackLand, Moscow, Russia) material using a flat horizontal surface at a distance of 5–7 cm. After being sprayed, the diameter of the colored circle was measured on the non-woven material.

#### 2.4.2. In Vivo “Animal” Studies

##### Animals

Fifty-four Wistar-type rats (54 males) weighing 270–330 g were utilized. The rats exploited in this study were procured from the “Andreevka” branch office of the Scientific Center for Biomedical Technologies of the Federal Medical and Biological Agency of Russia. The animals were maintained in standard laboratory conditions with controlled environmental parameters (12 h light–dark cycle, temperature 22 ± 2 °C, relative humidity 45–65%). The animals were provided with ad libitum access to food and water. Prior to the commencement of the study, they were allowed a one-week period of acclimatization.

All manipulations were conducted in accordance with the International Guiding Principles for Biomedical Research Involving Animals (EEU, Strasbourg, 1985), the European Convention for the Protection of Vertebrate Animals used for Experimental and Other Scientific Purposess (EEU, Strasbourg, 1986), the Guide for the Care and Use of Laboratory Animals (ILAR, DELS), the Rules of Laboratory Practice and the Russian Federation Ministry of Health Order No. 199n (1 April 2016), and “On Approval of the Rules of Laboratory Practice”, the relevant procedures were employed.

The animal study protocol was approved by the Ethics Committee of Preclinical Research Center, Sechenov First Moscow State Medical University of the Ministry of Health of the Russian Federation (Sechenov University) (protocol No. 149 from 13.11.2023).

The animals were randomly assigned to groups according to the time points (Table 3).

##### Sample Collection

Following administration, cardiac puncture was carried out on anesthetized rats at specific time intervals, with blood collected into vacuum lithium–heparin Bodywin^®^ tubes (Shandong Weigao, Weihai, China). Subsequently, the blood samples were centrifuged at 3500 rpm for 15 min (g = 1507). The resulting supernatant was transferred to Eppendorf tubes and then frozen at −20 °C for subsequent pharmacokinetic analysis. The brain and olfactory bulbs were harvested post-total body perfusion with cold PBS at pH 7.4 to eliminate any residual blood [53]. The brain and olfactory bulb samples were then frozen at −20 °C for further analysis.

Upon thawing, the olfactory bulbs and brains were weighed and then homogenized using a FASTPREP-24 homogenizer (Mp Biomedicals Germany GmbH, Eschwege, Germany) in a 0.9% NaCl solution. The used ratio (*w*/*w*) was 1:1 for brains and 1:20 for olfactory bulbs. This homogenization process was repeated three times for 20 s each at a speed of 4.0 m/s. Subsequently, the homogenate was centrifuged under the following conditions: 20 min at 15,000 rpm (g = 27,671) for the brain homogenate and 15 min at 1500 rpm (g = 277) for the olfactory bulb homogenate. The resulting supernatant was then transferred to Eppendorf tubes and stored at −20 °C until ready for pharmacokinetic analysis.

##### Pharmacokinetic Study

Samples were prepared for instrumental analysis by protein precipitation. To a 50 µL aliquot, 450 µL of acetonitrile was added, and the resulting solution was gently mixed on an orbital shaker for 30–40 s. The tubes containing the samples were then centrifuged at 13,000 rpm (g = 20,784) for 5 min. After centrifugation, 400 μL of the supernatant was collected, transferred to an Eppendorf tube, and evaporated to dryness, and the residue was redissolved in 100 μL of water and sent for pharmacokinetic analysis.

This study was performed on an Agilent 1290 Infinity II high-pressure liquid chromatograph (Agilent Technologies^®^, USA) with an Agilent 6470 Triple Quad LC/MS tandem quadrupole mass spectrometric detector (Agilent Technologies^®^, USA). Chromatography was performed in gradient elution mode (Table 4). Mobile phase A consisted of 0.1% formic acid, 0.01% heptafluorobutyric acid, and 5 mM ammonium formate in aqueous medium. Mobile phase B consisted of a 0.1% solution of formic acid, 0.01% heptafluorobutyric acid, and 5 mM ammonium formate in acetonitrile. Analysis was performed on a Discovery^®^ HS F5-3 15 cm × 2.1 mm, 3 µm Supelco reverse phase column at 40 °C with a mobile phase flow rate of 0.4 mL/min. The analysis time for each sample was 8 min. The injected sample volume was 5 µL. The detector parameters are shown in Table 5.

The initial chromatographic–mass spectrometric data were processed using the MassHunter software (version 12.0) package from Agilent Technologies (USA). The integration of chromatographic peaks was performed in automatic mode.

The method was validated for selectivity, linear range, accuracy, reproducibility, stability of the analyte in the solution and matrix, extraction degree, and matrix effect according to ICH Guideline M10 on bioanalytical method validation. More information concerning the method validation parameters is presented in Appendix A.

##### Statistical Analysis

Statistical analysis was performed using SPSS 27.0.1.0 for Windows (IBM Analytics, USA). Data are presented as mean ± SD (M ± SD). Comparison between different time points within each of the biosamples in experimental and control groups was performed using two-way ANOVA analysis of variance followed by Tukey’s post hoc test. Student’s *t*-test was used to compare concentrations in brain and olfactory bulb samples within similar time points. Differences between samples were considered statistically significant at a pre-determined significance level of *p* < 0.05.

## 3. Results

### 3.1. Preparation of Stimulus-Sensitive Compositions

A total of 64 placebo samples were prepared for in vitro studies of stimulus-sensitive compositions (Table 6). Depending on the phase transition stimulus, the sample preparation technology varied.

The thermoreversible compositions were prepared using the cold method at room temperature and mixed on a magnetic stirrer. After preparation, the compositions were sent for stabilization in a refrigerator at 2–8 °C after the structuring time. The thermoreversible placebo compositions were stored in the same temperature range (2–8 °C).

Ion-selective compositions were prepared by stirring on a magnetic stirrer with heating up to 80 °C. After complete swelling and dissolution of the polymers, the compositions were sent for stabilization in a refrigerator at a temperature of 2–8 °C after the structuring time. Placebo compositions were stored in the same temperature range (2–8 °C). The pH-sensitive formulations with Carbopol in the formulation did not require heating and were prepared cold by stirring on a magnetic stirrer until the polymer swelled and dissolved.

Since the chitosan-containing compositions were presumed to have the ability of thermoreversible and pH-sensitive phase transition, they were prepared using the cold method at room temperature and stirred on a magnetic stirrer. After preparation, the formulations were sent to a refrigerator at 2–8 °C for stabilization after the structuring time. Storage of these formulations was carried out under the same conditions (2–8 °C).

All in vitro studies were performed 12 h after preparation of the formulations.

### 3.2. In Vitro Studies Results

#### 3.2.1. Gelation Ability of Placebo Compositions

After preparation and structuring of the samples, the studies were conducted in the logical order determined by the design of the experiment (Figure 1). First, the gelling ability of the placebo formulations was evaluated (Table 7). Since this study allows for immediate identification of potentially suitable formulations for intranasal delivery, it is the first in a series of studies.

At this stage, the suitability of various thermoreversible, pH-sensitive, and ion-selective compositions can be concluded. When the compositions were immersed in nasal cavity conditions, only 10 out of 64 compositions did not undergo an in situ phase transition (37, 40, 55, 56, 57, 58, 59, 61, 63, 64). The remaining compositions showed at least partial gelation under physiological conditions of the nasal cavity.

#### 3.2.2. pH of Placebo Compositions

The next study was to determine the pH of the test formulations. The goal of the test compositions was to approximate the physiological pH of the nasal cavity. A critical deviation from the physiological pH often leads to ciliotoxic effects on the nasal mucosa. Table 8 summarizes the results of the tests performed.

The data obtained only confirmed that non-neutralized carbopol is too acidic to be used in nasal dosage form technology. All compositions containing this polymer were found to be more acidic than the mucosal surface. Compositions with such pH values (49–54) are likely to cause irritation on the mucosal surface.

#### 3.2.3. Plastic Viscosity of Placebo Compositions

In addition to being a technological indicator of the dosage form, viscosity is also an indirect pharmacokinetic property of the drug. Bioavailability and spray pattern are directly dependent on viscosity. Formulations with high viscosity (>0.1 Pa·s) are unable to disperse in the nasal cavity and release the drug completely.

Formulations containing pectin (59, 60) and guar gum (37–40) as excipients had critically high viscosity according to the rheological analysis results (Table 9). These excipients were added to the formulations to enhance the ion-selective properties of gellan gum, but they only increased the viscosity and interfered with the completeness of the sol–gel phase transition.

#### 3.2.4. Gelation Temperature

Table 10 presents the findings of supplementary studies conducted only on the thermoreversible compositions. The optimal gelation temperature was selected to be below 32 °C in order to prevent premature gelation outside the application site and to facilitate phase transition under nasal cavity conditions.

A general study was also conducted to evaluate the gelation capability of all placebo formulations (both thermoreversible and ion-selective) at 32 °C (±0.1) (Table 11). As expected, only 11 formulations demonstrated gelation temperature values suitable for the requirements.

The primary outcome of the test was the confirmation of the absence of thermoreversible properties in the placebo formulation 23–60.

In this present study, it was assumed that compositions 1–22 exhibited thermoreversible properties. In compositions 17, 18, 21, and 22, the phase transition was initiated at a temperature lower than that programmed by the design of experiment.

#### 3.2.5. Completeness of Retention of Placebo Formulations

The most evident indicator of the suitability of a delivery system is the degree of retention observed on the nasal cavity surface. Due to the difficulty of performing such in vivo tests, a 3D model of the nasal cavity is a valuable alternative. A successful trial was defined as the achievement of complete retention of the dosage form above 80% of the injected volume.

The data obtained from Table 12 indicate that only 10 out of 60 formulations (15, 16, 27–29, 31, 32, 46–48) demonstrate satisfactory retention on the surface of the nasal cavity mucosa. A significant number of these formulations undergo phase transition upon exposure to nasal discharge ions, which are ion-selective. Only two thermoreversible compositions exhibited retention above 80%—15 and 16.

#### 3.2.6. Spray Torch of Placebo Formulations

A narrow spray torch with a diameter of less than six centimeters is insufficient for the complete distribution of the system over the mucosal surface. Despite the objective of delivering the system to the olfactory bulb, systems with a narrow spray torch will be rapidly removed from the mucosal surface due to the large particle size of the atomized particles. However, a spray pattern exceeding 10 cm would also be inappropriate in the context of developing the delivery system, as the drug would not reach the olfactory bulb and therefore would not reach the brain (Table 13).

As described earlier, the viscosity of the composition directly affects the spray pattern. Thus, the compositions 19, 21, 22, 37–39, 49, 58, and 59 had very narrow atomization flares (<6 cm) due to their relatively high viscosity.

#### 3.2.7. Results of In Vitro Studies after API Addition

Following the analysis of the data collected during the examination of placebo compositions, the ones that met the CQA standards were chosen utilizing the MiniTab 17.0 software. It was observed that temperature (15, 16) and the ionic composition of nasal discharge (27, 28, 31, 32, 46–48) served as triggers for in situ gelation in these selected compositions. To assess the potential impact of API on the in situ gel performance, the same series of tests conducted on the placebo formulations (gel formation, pH, viscosity, gelation temperature, retention, and spray torch) were carried out on a specific group of nine formulations selected in the previous phase (Table 14).

Composition № 31, the ultimate leader in the series, with a composition of 0.5% GG and 2% P124, was identified through analysis using the MiniTab 17.0 software based on the obtained outcomes. All quality parameters fall within the desired ranges, and the formulation’s retention capacity improved since the API was introduced. It is plausible to suggest that the Poloxamer 124 in this blend exhibits mucoadhesive characteristics, facilitating the composition’s optimal adhesion to the mucosal surface. As a result, this formulation has been selected for additional examination in animal models.

### 3.3. In Vivo “Animal” Studies Results

#### 3.3.1. Analytical Study of Brain, Olfactory Bulb, and Blood Plasma Samples

**Figure 2:**
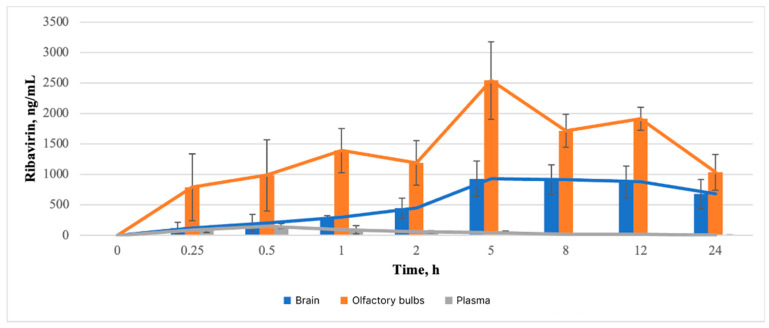
The concentration-time profiles of ribavirin in plasma, brain, and olfactory bulb samples were evaluated following intranasal administration to rats in the form of an in situ gel (experimental group). The data are presented as mean ± SD (M ± SD), with n = 3. Pharmacokinetic curves are presented in average format.

**Figure 3:**
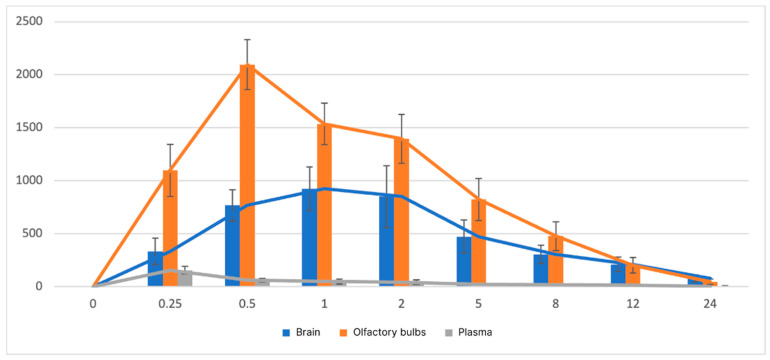
The concentration-time profiles of ribavirin in plasma, brain, and olfactory bulb samples were evaluated following intranasal administration to rats in the form of a water solution (control group). The data are presented as mean ± SD (M ± SD), with n = 3. Pharmacokinetic curves are presented in average format.

As previously described, the animals were administered a composition numbered 31 that met all in vitro characteristics. This composition included 0.5% GG and 2% P124. Figure 2 and Figure 3 depict the ribavirin concentration-time profiles in plasma, brain, and olfactory bulb samples following intranasal administration in experimental and control groups, respectively.

Table 15 presents the pharmacokinetic parameters obtained from the pharmacokinetic analysis of ribavirin distribution in brain, plasma, and olfactory bulb samples in experimental and control groups.

#### 3.3.2. Pharmacokinetic Study of Brain

In the study of brain samples of the experimental group, no significant differences were observed in the concentration between the time points of 15 min, 30 min, one hour, and two hours (*p* > 0.05). Consequently, in comparison to the aforementioned time points of 15 min, 30 min, and 1 h, statistically significant alterations in concentration levels were observed at the subsequent time points of 5 h, 8 h, and 12 h (*p* < 0.05), indicating an increase in the concentration of ribavirin in the brain during the aforementioned 5 h period. Additionally, no significant statistical differences were observed at the 5 h, 8 h, 12 h, and 24 h time points, suggesting that high brain concentrations of ribavirin are maintained for at least 19 h (5–24 h) when it is administered in the form of in situ nasal gel. Moreover, statistically significant differences (*p* < 0.05) were observed when comparing concentrations at 15 min and 24 h. This may indicate that high ribavirin concentrations in the brain are maintained for a longer period than at the maximum point studied, consistent with the MRT-inf_obs value (Figure 2).

From the study of the control group, it can be seen that the differences between the time points of 0.5, 1, and 2 h are not statistically significant (*p* > 0.05). At the same time, statistically significant differences are visible between the above-mentioned time points and time points of 0.25, 8, 12, and 24 h (*p* < 0.05). It can be said that with intranasal administration of ribavirin to rats in the form of an aqueous solution, the concentration of the drug reaches its peak within 30 min, remains approximately at the same level for an hour and a half, and then begins to decrease rapidly by 8 h after administration (Figure 3).

#### 3.3.3. Pharmacokinetic Study of Olfactory Bulb

As with the brain samples, when the olfactory bulb samples were examined, there were no significant differences in concentration between the time points of 15 min, 30 min, one hour, two hours, and the zero point (*p* > 0.05). Additionally, no statistically significant differences were observed in concentration levels at the 5 h, 8 h, and 12 h time points (*p* > 0.05). The three main points of comparison for drug concentrations were identified: 15 min, 5 h, and 24 h. Statistically significant changes in concentrations were recorded at each of these time points (*p* < 0.05), indicating an increase in ribavirin concentrations in olfactory bulbs within 5 h after administration, maintenance of the same concentration level up to 12 h, and a decrease in concentration by 24 h. Conversely, no statistically significant differences were observed between the points of 15 min, 24 h, and the zero point (*p* > 0.05), indicating a significant decrease in the concentration of ribavirin in olfactory bulbs to the point of 24 h after administration. Moreover, at all tested points except for 15 min and 24 h, the concentration of ribavirin in the olfactory bulbs was found to be higher than that in the brains (*p* < 0.05). At 24 h, no statistically significant differences were observed between ribavirin concentrations in the olfactory bulbs and brains (*p* > 0.05), which may indicate the potential role of olfactory bulbs in this method of administration as a carrier of the drug in the brain (Figure 2).

When examining the olfactory bulbs of rats from the control group, it becomes clear that the concentration of ribavirin rapidly increases and reaches its peak 30 min after administration. Also, Figure 3 shows that by 5 h after administration, the concentration of ribavirin, compared with the time point 0.5, drops by about two times and continues to decrease rapidly by the eighth, twelfth, and twenty-fourth time points (*p* < 0.05).

#### 3.3.4. Pharmacokinetic Study of Blood Plasma

Upon examination of the plasma samples, no statistically significant differences in ribavirin concentration levels were observed between points 2, 5, 8, 12, and 24 h and the zero point (*p* > 0.05). Concurrently, statistically significant alterations were observed between the aforementioned points and the 30 min point (*p* < 0.05), which suggests a rapid increase in ribavirin concentration in the plasma (within 30 min) and a rapid elimination of the drug from the plasma (Figure 2).

The maximum concentration of ribavirin in the plasma of rats in the control group is observed 15 min after administration. In the experimental group, the T_max_ value falls at the 0.5 point (Table 15). This may serve as additional confirmation that the intranasal in situ gel provides modified ribavirin release.

The low values of C_max_, AUC_0-t_, and AUC_0-inf_ in plasma, in comparison with the values of these parameters in brains and olfactory bulbs (Table 15), may indicate a small degree of passage of ribavirin into the general bloodstream through the capillary network of the nasal cavity. This apparent passage is likely due to the polar nature of the substance [54].

## 4. Discussion

Consistent and thorough analysis of all in situ-generated compositions with different gelation stimuli led to certain results that allowed us to proceed to in vivo studies.

The initial screening of placebo compositions permitted the formation of conclusions regarding the suitability of certain compositions for further development. Despite the potential for phase transition in some of the compositions, their gelation did not occur in accordance with the physiological conditions of the nasal cavity, thus precluding their classification as leading compositions.

As part of the implementation of pharmaceutical development according to QbD standards, the dependence of quality attributes on process parameters (CQA/CPP) was also evaluated. Table 16 presents a summary of the results, which demonstrates the correlation between CQA and CPP.

It has been established that the only process parameter that affects CQA is the temperature at which the composition is made. This parameter can have a detrimental effect on thermoreversible compositions that require only cold preparation. Furthermore, the manufacture of compositions with polymers that require heating during dissolution should be conducted in a specific temperature regime, which will ensure the production of a product of satisfactory technological characteristics.

In addition to process parameters, the process characteristics can be influenced by the materials (polymers) themselves and additional auxiliary substances.

The results of all tests demonstrated that the presence of additional excipients and their concentration exerted a significant influence on the characteristics of the final delivery system (Table 17). In this case, the concentration of the active pharmaceutical ingredient (API) exerts a relatively minor influence on the final quality of the drug. The optimal ratio of smart polymer and additional excipient enabled the development of an innovative stimulus-sensitive system for intranasal delivery of ribavirin directly from the nose to the brain.

Pharmacokinetic parameters of ribavirin obtained in the study of brains and olfactory bulbs show that when the drug is administered intranasally in the form of an in situ gel, the concentration of ribavirin in this tissue is retained significantly longer than when ribavirin is administered in the form of an aqueous solution (Table 15). It is assumed that the concentration of ribavirin in brains and olfactory bulbs at the peak point of the experimental group is no less than the values of the control group.

The high values of C_max_, AUC_0-t_, and AUC_0-inf_ in olfactory bulb and brain samples, in conjunction with their low values in plasma, indicate that the drug in question is transported from the nose to the brain via olfactory neuronal pathways, bypassing the BBB [54]. This finding is consistent with previous studies that have demonstrated the transport of ribavirin via the nose-to-brain pathway [55].

The values of t_1/2_ and MRT_0-inf_obs_ of ribavirin for olfactory bulbs and brains in the experimental group compared with the control group indicate a prolonged release of this drug when used as an intranasal in situ gel. When ribavirin is administered in gel form, the concentration of the drug in the brain can be maintained for at least 19 h (5–24 h), while in the case of olfactory bulbs, the concentration remains elevated for at least 7 h (5–12 h). A gradual increase in concentration in the brain and olfactory bulbs for five hours before reaching the maximum concentration, compared with a more intensive achievement of ribavirin in brain and olfactory bulbs tissues when used as an aqueous solution, also indicates a modified release of ribavirin in the form of an in situ gel. Higher values of t_1/2_ and MRT_0-inf_obs_ in the experimental group, observed in brain samples compared with olfactory bulb samples, may indicate that the olfactory bulb serves as an “intermediate site” for ribavirin delivery to the brain.

## 5. Conclusions

Following an extensive in vitro quality analysis of 60 stimulus-sensitizing placebo formulations, one formulation was selected that met all characteristics. This compound contained 0.5% GG and 2% P124 and was acceptable for further pharmacokinetic studies in animal models.

This article is the first to report the pharmacokinetic characterization of ribavirin in the brain, olfactory bulbs, and plasma after its intranasal administration in the form of an in situ gel in comparison with administration in the form of a water solution. Based on the results obtained, it can be concluded that due to low concentrations of ribavirin in plasma on the one hand, and high content of the drug in olfactory bulbs on the other hand, intranasally administered in situ gel with ribavirin specifically reached the CNS via olfactory pathways, and only a small part of the drug entered the systemic circulation. In addition to the demonstrated low rate of passage of ribavirin into the systemic circulation in this dosage form, potentially reducing the likelihood of systemic side effects, and its prolonged action, potentially increasing the efficacy of ribavirin in the intranasal in situ gel form, the findings of Said and Elmenoufy of reduced systemic side effects, as well as the increased efficacy and bioavailability of the drugs in the form of modified-release dosage forms, show that the drug we are investigating is promising for pharmaceutical development using the QbD approach [56].

The proposed QbD pharmaceutical development design identified a formulation containing 0.5% gellan gum and 2% Poloxamer 124 as the optimal composition with ion selectivity towards artificial nasal fluid. After introducing ribavirin into the composition, it showed satisfactory in vitro performance. The data obtained allowed for animal studies to be performed. The results of the in vivo studies proved the existence of the mechanism of drug transport from the nasal cavity by a nose-to-brain mechanism. The main opportunity for further research is to improve ethics by reducing the need to euthanize animals at each time point. By using the latest techniques of microdialysis of internal organs, it is possible to increase the speed of research and remove any doubts about the ethics of animal research.

## Figures and Tables

**Table 1:** Quality target product profile (QTPP).

Factor	Target	Justification
Route of administration	Intranasal	Directed delivery to the brain
Site of effect	Brain	Transport of AFI by cranial nerves (olfactory and trigeminal) to the brain
API administration	100 mg/mL	Test concentration with antitumor effect
pH	5.5–7.5	Optimal range for comfortable administration
Viscosity before gelation	Below 100 mPa·s	Phase transition is due to the selected nebulization system—NEST Pre-filled Disposable Intranasal Atomization Device (China)
Gelation stimulus	Temperature, ions (Na^+^, K^+^, Ca^2+^)	Gel formation conditions correspond to the physiologic parameters of the nasal cavity
Phase transition temperature	Below 32 °C	Nasal cavity temperature (applicable for thrmoreversible systems)
Spray torch	6–9 cm	Targeting the olfactory bulb
Retention on nasal cavity model	>80%	Similar retention percentage to highly adhesive compositions

**Table 2:** Critical parameters list.

CQA	CMA	CPP
Gelation stimulus	Smart-polymer concentration	Stirring time
Solution viscosity	Additional ingredient	Dispergating speed
Spray torch	Concentration of additional ingredient	Dispergating time
pH	Type of solvent	Manufacturing temperature
Retention on nasal cavity model	Concentration of API	

**Table 3:** Animal distribution by groups.

Animals	Groups	Number of Animals	Drug/Dose/Administration Volume	Manipulations
Rats, intranasal administration	0 min	3	In situ gel with ribavirin, 10 mg per kg, 15 µL in each nostril per 300 g (rat weight)	Vital manipulations—intranasal injection, blood sampling. After euthanasia—collection of brain and olfactory bulbs with further homogenization and analytical study.
	15 min	3		
	30 min	3		
	1 h	3		
	2 h	3		
	5 h	3		
	8 h	3		
	12 h	3		
	24 h	3		
**Control group**				
Rats, intranasal administration	0 min	3	Ribavirin water solution, 10 mg per kg, 15 µL in each nostril per 300 g (rat weight)	Vital manipulations—intranasal injection, blood sampling. After euthanasia—collection of brain and olfactory bulbs with further homogenization and analytical study.
	15 min	3		
	30 min	3		
	1 h	3		
	2 h	3		
	5 h	3		
	8 h	3		
	12 h	3		
	24 h	3		

**Table 4:** Gradient elution program.

Time, min	“A” Phase Amount, %	“B” Phase Amount, %
0	100	0
1.5	100	0
2.0	10	90
6.0	10	90
6.10	100	0
8.0	100	0

**Table 5:** Tandem quadrupole mass spectrometry detector parameters.

Parameter	Value
Ionization type	Electrospraying with heated atomizing gas stream
Gas Temp	300 °C
Gas Flow	11 L/min
Nebulizer	35 psi
SheathGasHeater	300 °C
SheathGasFlow	11 L/min
Capillary Voltage	2.5 kV
Mode of Analysis	MRM, positive ion

**Table 6:** Placebo compositions and polymer concentrations.

№	P407	P188	P124	GG	Ch	PBS	XG	GuG	PVA	HPMC	Pec	Carb	PEG 1500
**1**	16	3	-	-	-	-	-	-	-	-	-	-	-
**2**	16	2	-	-	-	-	-	-	-	-	-	-	-
**3**	18	3	-	-	-	-	-	-	-	-	-	-	-
**4**	18	2	-	-	-	-	-	-	-	-	-	-	-
**5**	16	-	2	-	-	-	-	-	-	-	-	-	-
**6**	16	-	1	-	-	-	-	-	-	-	-	-	-
**7**	18	-	2	-	-	-	-	-	-	-	-	-	-
**8**	18	-	1	-	-	-	-	-	-	-	-	-	-
**9**	16	-	-	-	-	-	-	-	-	-	-	-	1
**10**	16	-	-	-	-	-	-	-	-	-	-	-	0.5
**11**	18	-	-	-	-	-	-	-	-	-	-	-	1
**12**	18	-	-	-	-	-	-	-	-	-	-	-	0.5
**13**	16	-	-	-	2	-	-	-	-	-	-	-	-
**14**	16	-	-	-	1	-	-	-	-	-	-	-	-
**15**	18	-	-	-	2	-	-	-	-	-	-	-	-
**16**	18	-	-	-	1	-	-	-	-	-	-	-	-
**17**	16	-	-	-	-	-	-	-	-	-	-	-	-
**18**	18	-	-	-	-	-	-	-	-	-	-	-	-
**19**	16	-	-	0.25	-	-	-	-	-	-	-	-	-
**20**	16	-	-	0.5	-	-	-	-	-	-	-	-	-
**21**	18	-	-	0.25	-	-	-	-	-	-	-	-	-
**22**	18	-	-	0.5	-	-	-	-	-	-	-	-	-
**23**	-	-	-	0.25	-	-	-	-	-	-	-	-	-
**24**	-	-	-	0.5	-	-	-	-	-	-	-	-	-
**25**	-	-	-	0.25	-	10	-	-	-	-	-	-	-
**26**	-	-	-	0.25	-	15	-	-	-	-	-	-	-
**27**	-	-	-	0.5	-	10	-	-	-	-	-	-	-
**28**	-	-	-	0.5	-	15	-	-	-	-	-	-	-
**29**	-	-	2	0.25	-	-	-	-	-	-	-	-	-
**30**	-	-	1	0.25	-	-	-	-	-	-	-	-	-
**31**	-	-	2	0.5	-	-	-	-	-	-	-	-	-
**32**	-	-	1	0.5	-	-	-	-	-	-	-	-	-
**33**	-	-	-	0.25	-	-	0.25	-	-	-	-	-	-
**34**	-	-	-	0.25	-	-	0.5	-	-	-	-	-	-
**35**	-	-	-	0.5	-	-	0.25	-	-	-	-	-	-
**36**	-	-	-	0.5	-	-	0.5	-	-	-	-	-	-
**37**	-	-	-	0.25	-	-	-	0.25	-	-	-	-	-
**38**	-	-	-	0.25	-	-	-	0.5	-	-	-	-	-
**39**	-	-	-	0.5	-	-	-	0.25	-	-	-	-	-
**40**	-	-	-	0.5	-	-	-	0.5	-	-	-	-	-
**41**	-	-	-	0.25	-	-	-	-	0.25	-	-	-	-
**42**	-	-	-	0.25	-	-	-	-	0.5	-	-	-	-
**43**	-	-	-	0.5	-	-	-	-	0.25	-	-	-	-
**44**	-	-	-	0.5	-	-	-	-	0.5	-	-	-	-
**45**	-	-	-	0.25	-	-	-	-	-	0.1	-	-	-
**46**	-	-	-	0.25	-	-	-	-	-	0.2	-	-	-
**47**	-	-	-	0.5	-	-	-	-	-	0.1	-	-	-
**48**	-	-	-	0.5	-	-	-	-	-	0.2	-	-	-
**49**	-	-	-	0.25	-	-	-	-	-	-	-	0.1	-
**50**	-	-	-	0.25	-	-	-	-	-	-	-	0.2	-
**51**	-	-	-	0.5	-	-	-	-	-	-	-	0.1	-
**52**	-	-	-	0.5	-	-	-	-	-	-	-	0.2	-
**53**	-	-	-	-	-	-	0.25	-	-	-	-	-	-
**54**	-	-	-	-	-	-	0.5	-	-	-	-	-	-
**55**	-	-	-	0.25	-	-	-	-	-	-	0.1	-	-
**56**	-	-	-	0.25	-	-	-	-	-	-	0.2	-	-
**57**	-	-	-	0.5	-	-	-	-	-	-	0.1	-	-
**58**	-	-	-	0.5	-	-	-	-	-	-	0.2	-	-
**59**	-	-	-	-	-	-	-	-	-	-	0.25	-	-
**60**	-	-	-	-	-	-	-	-	-	-	0.5	-	-

All concentrations are given in percentages.

**Table 7:** Gel strength during in situ gelation of placebo formulations.

№	Gelation ^1^	№	Gelation ^1^	№	Gelation ^1^	№	Gelation ^1^
**1**	+++	**17**	+++	**33**	+	**49**	++
**2**	+++	**18**	+++	**34**	++	**50**	++
**3**	+++	**19**	+++	**35**	++	**51**	+++
**4**	+++	**20**	+++	**36**	++	**52**	+++
**5**	+++	**21**	+++	**37**	-	**53**	-
**6**	+++	**22**	+++	**38**	+	**54**	-
**7**	+++	**23**	++	**39**	+	**55**	-
**8**	+++	**24**	+++	**40**	-	**56**	+
**9**	+++	**25**	++	**41**	+	**57**	-
**10**	+++	**26**	++	**42**	+	**58**	+
**11**	+++	**27**	+++	**43**	++	**59**	-
**12**	+++	**28**	+++	**44**	++	**60**	+
**13**	+++	**29**	++	**45**	++		
**14**	+++	**30**	++	**46**	++		
**15**	+++	**31**	+++	**47**	+++		
**16**	+++	**32**	+++	**48**	+++		

^1^ For ease of interpretation of the results in Table 7, the data are labeled with symbols, where “-”, no gel forms; “+”, unstructured gel forms; “++”, solid gel forms, disintegrates within 10 min; and “+++”, solid gel forms, retains structure for more than 1 h.

**Table 8:** pH values of placebo compositions.

№	pH Value	№	pH Value	№	pH Value	№	pH Value
**1**	6.32	**17**	7.52	**33**	7.21	**49**	4.32
**2**	6.71	**18**	8.04	**34**	7.11	**50**	4.21
**3**	6.34	**19**	7.92	**35**	7.3	**51**	4.15
**4**	6.51	**20**	8.1	**36**	6.99	**52**	4.25
**5**	6.96	**21**	7.89	**37**	6.76	**55**	6.75
**6**	6.84	**22**	7.3	**38**	6.6	**56**	6.88
**7**	6.89	**23**	6.93	**39**	6.89	**57**	6.92
**8**	7	**24**	6.9	**40**	6.91	**58**	7.07
**9**	6.7	**25**	6.95	**41**	6.54	**59**	7.04
**10**	6.44	**26**	7.02	**42**	6.77	**60**	6.89
**11**	6.58	**27**	7.14	**43**	6.72		
**12**	6.7	**28**	7.21	**44**	6.81		
**13**	6.51	**29**	7.04	**45**	7.49		
**14**	6.1	**30**	6.98	**46**	7.62		
**15**	6.12	**31**	6.82	**47**	6.44		
**16**	6.3	**32**	6.91	**48**	6.58		

**Table 9:** Placebo composition plastic viscosity according to the Casson model.

№	Viscosity, mPa·s	№	Viscosity, mPa·s	№	Viscosity, mPa·s	№	Viscosity, mPa·s
**1**	45.6	**17**	21.2	**33**	40.1	**49**	27.4
**2**	41.3	**18**	29.2	**34**	32.2	**50**	27.5
**3**	69.1	**19**	59.9	**35**	40.1	**51**	28.7
**4**	62.4	**20**	59.1	**36**	75.3	**52**	29.9
**5**	45.7	**21**	53.3	**37**	138.9	**55**	22.2
**6**	42	**22**	84.5	**38**	134.2	**56**	29.7
**7**	82.6	**23**	40.5	**39**	129.9	**57**	69.7
**8**	32.8	**24**	61.1	**40**	131.2	**58**	94.6
**9**	28.2	**25**	56.8	**41**	59.8	**59**	115.1
**10**	24.3	**26**	54.8	**42**	59.7	**60**	106.9
**11**	29.3	**27**	30.1	**43**	54.1		
**12**	27.2	**28**	33.3	**44**	58.2		
**13**	41	**29**	27.1	**45**	39.8		
**14**	39.8	**30**	28	**46**	42.2		
**15**	42.3	**31**	27.8	**47**	39.7		
**16**	40.9	**32**	59.8	**48**	45.6		

**Table 10:** Gelation temperature of placebo formulations.

№	Gelation t, °C	№	Gelation t, °C
**1**	31.2	**12**	36.6
**2**	30.9	**13**	36
**3**	39.9	**14**	31.1
**4**	33.1	**15**	28.1
**5**	32.3	**16**	29.2
**6**	34.7	**17**	27.6
**7**	35.3	**18**	28.5
**8**	31.2	**19**	36.4
**9**	31.4	**20**	32.1
**10**	35	**21**	27.4
**11**	34.6	**22**	28.3

**Table 11:** Gelation temperature of placebo formulations at temperature 32 °C (SD ± 0.1).

№	Gelation t, °C ^1^	№	Gelation t, °C ^1^	№	Gelation t, °C ^1^	№	Gelation t, °C ^1^
**1**	+	**17**	+	**33**	-	**49**	-
**2**	+	**18**	+	**34**	-	**50**	-
**3**	-	**19**	-	**35**	-	**51**	-
**4**	-	**20**	-	**36**	-	**52**	-
**5**	-	**21**	+	**37**	-	**53**	-
**6**	-	**22**	+	**38**	-	**54**	-
**7**	-	**23**	-	**39**	-	**55**	-
**8**	+	**24**	-	**40**	-	**56**	-
**9**	+	**25**	-	**41**	-	**57**	-
**10**	-	**26**	-	**42**	-	**58**	-
**11**	-	**27**	-	**43**	-	**59**	-
**12**	-	**28**	-	**44**	-	**60**	-
**13**	-	**29**	-	**45**	-		
**14**	+	**30**	-	**46**	-		
**15**	+	**31**	-	**47**	-		
**16**	+	**32**	-	**48**	-		

^1^ For ease of interpretation of the results in Table 11, the data are labeled with symbols, where “-”, no gel forms; “+”, gel forms at temperature 32 °C.

**Table 12:** Completeness of retention of placebo formulations on the surface of an in vitro model of the nasal cavity (n = 5) (*p* = 0.05).

№	Retention, %	№	Retention, %	№	Retention, %	№	Retention, %
**1**	33	**17**	35	**33**	15	**49**	10
**2**	30	**18**	39	**34**	25	**50**	15
**3**	10	**19**	70	**35**	20	**51**	15
**4**	18	**20**	72	**36**	23	**52**	17
**5**	64	**21**	65	**37**	29	**53**	5
**6**	68	**22**	60	**38**	35	**54**	10
**7**	70	**23**	67	**39**	35	**55**	15
**8**	70	**24**	70	**40**	40	**56**	18
**9**	39	**25**	70	**41**	55	**57**	20
**10**	31	**26**	77	**42**	49	**58**	18
**11**	37	**27**	85	**43**	65	**59**	18
**12**	35	**28**	90	**44**	63	**60**	20
**13**	72	**29**	80	**45**	53	**61**	8
**14**	76	**30**	75	**46**	87	**62**	6
**15**	85	**31**	95	**47**	87	**63**	20
**16**	80	**32**	90	**48**	90	**64**	15

**Table 13:** Spray torch of placebo formulations (*p* = 0.05).

№	Spray Torch, cm	№	Spray Torch, cm	№	Spray Torch, cm	№	Spray Torch, cm
**1**	11	**17**	8	**33**	9	**49**	5
**2**	9	**18**	9	**34**	11	**50**	6
**3**	8	**19**	5.5	**35**	6	**51**	5.5
**4**	10	**20**	6	**36**	8	**52**	4.5
**5**	9.5	**21**	4	**37**	2.5	**53**	6
**6**	8.5	**22**	3.5	**38**	3	**54**	8
**7**	10	**23**	12	**39**	3	**55**	8.5
**8**	11	**24**	13.5	**40**	6.5	**56**	5
**9**	11	**25**	9	**41**	7.5	**57**	8
**10**	10.5	**26**	8.5	**42**	8.5	**58**	3
**11**	9.5	**27**	10	**43**	8	**59**	3.5
**12**	13	**28**	10.5	**44**	9	**60**	6
**13**	11	**29**	12	**45**	8.5		
**14**	9.5	**30**	11	**46**	7		
**15**	8.5	**31**	8	**47**	8		
**16**	9	**32**	7.5	**48**	9.5		

**Table 14:** Results of in vitro studies after addition of ribavirin (100 mg/mL).

With Ribavirin						
№	Gelation	Gelation t, °C	pH Value	Retention, %	Viscosity, mPa·s	Spray Torch
**15**	+++	25	6.12	45	27.1	12
**16**	+++	24.6	6.3	60	39.9	9
**27**	+++	-	7.14	80	37.8	9.5
**28**	+++	-	7.21	85	30.2	10
**31**	+++	-	6.82	98	28.1	8.5
**32**	+++	-	6.91	90	24.1	9
**46**	++	-	7.62	85	29.4	10.5
**47**	+++	-	6.44	83	35.1	7.5
**48**	+++	-	6.58	85	29.9	8
**Without ribavirin**						
**15**	+++	28.1	6.12	85	42.3	8.5
**16**	+++	29.2	6.3	80	40.9	9
**27**	+++	-	7.14	85	30.1	10
**28**	+++	-	7.21	90	33.3	10.5
**31**	+++	-	6.82	95	27.8	8
**32**	+++	-	6.91	90	59.8	7.5
**46**	++	-	7.62	87	42.2	7
**47**	+++	-	6.44	87	39.7	8
**48**	+++	-	6.58	90	45.6	9.5

For ease of interpretation of the results in Table 14, the data are labeled with symbols, where “-”, no gel forms; “++”, solid gel forms, disintegrates within 10 min; and “+++”, solid gel forms, retains structure for more than 1 h.

**Table 15:** Pharmacokinetic parameters of ribavirin in brain, plasma, and olfactory bulb samples after its administration to rats in experimental and control groups.

Parameter *	Unit	Plasma Value	Brain Value	Olfactory Bulbs Value
**Experimental group**				
Λ_z	1/h	0.1289	0.0335	0.0378
t1/2	h	5.3791	20.6782	18.3222
Tmax	h	0.5	5	5
Cmax	ng/ml	152.6586	931.1437	2542.7953
Tlag	h	0	0	0
Clast_obs/Cmax	-	0.0228	0.5890	0.4073
AUC 0-t	ng/mL*h	629.5281	17,535.4248	38,105.3782
AUC 0-inf_obs	ng/mL*h	656.5046	33,897.8019	65,483.2040
AUC 0-t/0-inf_obs	-	0.9589	0.5173	0.5819
AUMC 0-inf_obs	ng/mL*h^2^	4569.5688	1,085,744.771	1,796,570.896
MRT 0-inf_obs	h	6.9605	32.0299	27.4356
Vz/F_obs	(μg/kg)/(ng/mL)	0.1182	0.0088	0.0040
Cl/F_obs	(μg/kg)/(ng/mL)/h	0.0152	0.0003	0.0002
**Control group**				
Λ_z	1/h	0.0960	0.0837	0.1549
t1/2	h	7.2175	8.2803	4.4754
Tmax	h	0.25	1	0.5
Cmax	ng/ml	154.3622	924.4831	2062.3333
Tlag	h	0	0	0
Clast_obs/Cmax	-	0.0220	0.0857	0.0232
AUC 0-t	ng/mL*h	421.3711	7412.3988	11,029.7240
AUC 0-inf_obs	ng/mL*h	456.7740	8359.1850	11,338.1257
AUC 0-t/0-inf_obs	-	0.9225	0.8867	0.9728
AUMC 0-inf_obs	ng/mL*h^2^	3885.3060	85,566.0065	68,537.7786
MRT 0-inf_obs	h	8.5060	10.2361	6.0449
Vz/F_obs	(μg/kg)/(ng/mL)	0.2280	0.0143	0.0057
Cl/F_obs	(μg/kg)/(ng/mL)/h	0.0219	0.0012	0.0009

* Λ_z, apparent terminal elimination rate constant; t_1/2_, elimination half-life; T_max_, time to reach this plasma concentration; C_max_, maximum plasma concentration; Tlag, time between dosing and the start of absorption; C_last_obs_, last measurable positive concentration; AUC_0-t_, area under the plasma concentration-time curve; AUC_0-inf obs_, area under the zero-to-infinity curve; AUMC_0-inf obs_, area under the first-moment curve; MRT_0-inf_obs_, mean residence time; Vz/F__obs_, volume of distribution; Cl/F__obs_, total body clearance.

**Table 16:** Results of determining the dependence of CQA on CPP.

CQA/CPP	Stirring Time	Dispergating Speed	Dispergating Time	Manufacturing Temperature	Cross-Linking Time
**Gelation stimulus**	Low	Low	Low	High	Low
**Solution viscosity**	Medium	Low	Low	Low	Low
**Spray torch**	Low	Low	Low	Low	Low
**pH**	Low	Low	Low	Low	Low
**Retention on nasal cavity model**	Low	Low	Low	Low	Low

**Table 17:** Results of determining the dependence of CQA on CMA.

CQA/CMA	Smart-Polymer Concentration	Additional Ingredient	Concentration of Additional Ingredient	Type of Solvent	Concentration of API
**Gelation stimulus**	High	High	High	High	Low
**Solution viscosity**	High	High	High	High	Medium
**Spray torch**	High	High	High	High	Medium
**pH**	Medium	Medium	Medium	High	Medium
**Retention on nasal cavity model**	High	High	High	High	Medium

## Data Availability

The data presented in this study are openly available in the article.

## Appendix A

Validation parameters of quantitative analysis of ribavirin in different biological samples, Table A1: RT and MS/MS parameters, Table A2: Calibration curve parameters, Table A3: Within-batch precision and accuracy (n = 6) in blood plasma, Table A4: Between-batch precision and accuracy (n = 6) in blood plasma, Table A5: Within-batch precision and accuracy (n = 6) in brain, Table A6: Between-batch precision and accuracy (n = 6) in brain, Table A7: Within-batch precision and accuracy (n = 6) in olfactory bulb, Table A8: Between-batch precision and accuracy (n = 6) in olfactory bulb, Table A9: Stability of ribavirin in biological samples.

### Appendix A.1. Reagents

Ribavirin was obtained from Chimmed (Russia) and 13C5-ribavirin, cytidine, and uridine were obtained from Toronto Research Chemicals (Canada). The purity of the chemicals was “Pro Analysi”—pure for analytical studies. High-purity water was prepared in-house using a Milli-Q water purification system obtained from Merck KGaA (Darmstadt, Germany). HPLC-grade methanol and acetonitrile were purchased from Thermo Fisher Scientific Inc. (Madrid, Spain) and Panreac (Barcelona, Spain), respectively. Extra-pure formic acid, heptafluorobutyric acid, and ammonium formate were purchased from Fluka (Steinheim, Germany).

### Appendix A.2. Method

**Table A1:** RT and MS/MS parameters.

Analyte	Retention Time (min)	Precursor (m/z)	Product (m/z)	Fragmentor (V)	CE (V)
**Ribavirin**	1.57	245	113	80	7
**^13^C_5_-Ribavirin**	1.57	250	113	80	7
**Uridine**	2.01	245	113	60	5
**Cytidine**	2.33	244	112	60	5

### Appendix A.3. Selectivity

The selectivity and specificity of the method were examined by comparing mass chromatograms of blank blood plasma, brain, and olfactory bulb samples with quality control samples at LLOQ levels. The MRM transition for ribavirin coincides with uridine; isobaric endogenous compounds may influence the results of quantitative determination. Under these chromatographic conditions, chromatographic separation of ribavirin, cytidine, and uridine was achieved, which made it possible to distinguish the required selectivity and specificity of the procedure. The retention times were 1.57, 2.01, and 2.33 for ribavirin, uridine, and cytidine, respectively.

There was no effect on the retention time of ribavirin or IS and baseline noise levels did not exceed 20% of the relative peak area response of the analytes.

### Appendix A.4. Linearity

Calibration was performed using an isotopically labeled internal standard. The peak area ratios of the calibration standards were proportional to the concentration of the analytes. The calibration curve was linear over the concentration range 1–1000 ng/mL and was well described by a linear regression equation (Table A2). To achieve homogeneity of variance, a weighting factor of 1/x was applied. The correlation coefficient (r) was 0.9991. The calculated concentrations of the calibration standards were within 15% of their nominal values and ranged between 89.1 and 110.2%.

**Table A2:** Calibration curve parameters.

Analyte	Linear Range (ng/mL)	Slope Mean ± SD	Intercept Mean ± SD	R Mean ± SD
**Blood plasma**				
**Ribavirin**	1–1000	1.214 ± 0.017	0.065 ± 0.021	0.9991 ± 0.0002
**Brain**				
**Ribavirin**	1–1000	1.085 ± 0.045	0.047 ± 0.013	0.9989 ± 0.0005
**Olfactory bulb**				
	1–1000	1.101 ± 0.014	0.074 ± 0.005	0.9998 ± 0.0003

### Appendix A.5. Lowest Limit of Quantification

Analysis of LLOQ samples for three analytical runs revealed that a concentration level of 1 ng/mL can be quantified precisely and accurately. The accuracy levels for LLOQ for plasma samples were 87.7–92.6% between runs and 93.4% within runs, while the precision parameters were 0.88–2.67% between runs and 3.26% within runs. For brain samples, the accuracy levels for LLOQ were 86.9–105% between runs and 89.5% within runs, while for precision parameters they were 5.9–10.3% between runs and 5.4% within runs. The accuracy levels for LLOQ for olfactory bulb samples were 94.5–100.6% between runs and 97.1% within runs, while precision parameters were 1.2–3.3% between runs and 3.26% within runs.

### Appendix A.6. Accuracy and Precision

Quality control samples prepared at three concentration levels were analyzed in three analytical runs of six replicates each. The concentration of quality control samples was determined using linear regression parameters and compared with the theoretical value. The accuracy and precision results (Table A3, Table A4, Table A5, Table A6, Table A7 and Table A8) showed that the average concentration values were within 10% of the nominal values of the quality control samples, while the relative standard deviation did not exceed the threshold value of 15%.

**Table A3:** Within-batch precision and accuracy (n = 6) in blood plasma.

Analyte	Spiked Concentration (ng/mL)	Calculated Concentration (ng/mL)	RSD (%)	Accuracy, %
**Ribavirin**	3	3.17 ± 0.34	10.7	105.7
	300	310.1 ± 9.4	3.0	103.4
	750	765 ± 38	4.9	102.0

**Table A4:** Between-batch precision and accuracy (n = 6) in blood plasma.

Analyte	Spiked Concentration (ng/mL)	Calculated Concentration (ng/mL)	RSD (%)	Accuracy, %
**Ribavirin**	3	3.05 ± 0.14	4.6	101.7
	300	316.6 ± 7.2	2.3	105.5
	750	785 ± 21	2.7	104.7

**Table A5:** Within-batch precision and accuracy (n = 6) in brain.

Analyte	Spiked Concentration (ng/mL)	Calculated Concentration (ng/mL)	RSD (%)	Accuracy, %
**Ribavirin**	3	3.21 ± 0.42	13.1	107.0
	300	284.1 ± 22.5	7.91	94.7
	750	675 ± 54	8.0	90.0

**Table A6:** Between-batch precision and accuracy (n = 6) in brain.

Analyte	Spiked Concentration (ng/mL)	Calculated Concentration (ng/mL)	RSD (%)	Accuracy, %
**Ribavirin**	3	3.18 ± 0.11	3.5	106.0
	300	297.3 ± 13.5	4.5	99.1
	750	691 ± 47	6.8	108.9

**Table A7:** Within-batch precision and accuracy (n = 6) in olfactory bulb.

Analyte	Spiked Concentration (ng/mL)	Calculated Concentration (ng/mL)	RSD (%)	Accuracy, %
**Ribavirin**	3	3.01 ± 0.14	4.71	100.3
	300	307.2 ± 11.6	3.86	102.4
	750	849 ± 17	2.0	113.2

**Table A8:** Between-batch precision and accuracy (n = 6) in olfactory bulb.

Analyte	Spiked Concentration (ng/mL)	Calculated Concentration (ng/mL)	RSD (%)	Accuracy, %
**Ribavirin**	3	3.00 ± 0.12	1.05	100.1
	300	304.6 ± 9.6	3.2	101.5
	750	817 ± 26	3.1	108.9

### Appendix A.7. Recovery and Matrix Effect

The matrix effect was assessed using pre-prepared blood plasma, brain, and olfactory bulb samples, to which ribavirin was added to achieve concentrations corresponding to LQC and HQC (post-spike sample). The peak areas of ribavirin were compared with the standard. The matrix factor was 96–104% for blood plasma, 87–102% for the brain and 93–111% for the olfactory bulb. RSD did not exceed 15%.

Determination of the extraction recovery was carried out using samples with analyte concentrations corresponding to LQC and HQC prepared using blank blood plasma, brain, and olfactory bulb samples. The peak areas of ribavirin were compared in the chromatograms of samples to which ribavirin was added before and after extraction. The extraction recovery was 86–94% for blood plasma, 85–97% for the brain, and 94–97% for the olfactory bulb. RSD did not exceed 15%.

### Appendix A.8. Stability

The results of the stability experiments showed that there was no significant change in the concentration of ribavirin during chromatography, extraction, and storage of standards and biological samples. The stability of the processes was studied using LQC and HQC. The results showed that all analytes were stable in biological samples for 24 h at room temperature around 25 °C and 12 h in an autosampler at 6 °C. It was also confirmed that repeated freezing and thawing (five cycles) of spiked samples did not affect the stability of ribavirin. Stability data for all analytes are summarized in Table A9.

**Table A9:** Stability of ribavirin in biological samples.

Name	Sample	Concentration Level	Accuracy, %	Precision, %RSD
**Short-term stability in working solutions** **(RT, 6 h)**		LQC	95.1 ± 1.4	4.8
		HQC	94.8 ± 1.3	9.2
**Short-term stability ** **in biological samples (RT, 6 h)**	Plasma	LQC	87.4 ± 4.5	3.5
		HQC	101.9 ± 1.9	4.8
	Brain	LQC	98.4 ± 5.4	9.4
		HQC	102.4 ± 8.8	12.3
	Olfactory bulb	LQC	104.7 ± 0.8	11.8
		HQC	100.9 ± 1.7	10.9
**Autosampler stability ** **in biological samples ** **(+10 °C, 24 h)**	Plasma	LQC	104.8 ± 5.5	8.4
		HQC	104.2 ± 6.4	5.6
	Brain	LQC	100.1 ± 1.1	4.0
		HQC	97.8 ± 0.4	1.2
	Olfactory bulb	LQC	97.5 ± 4.8	5.9
		HQC	99.4 ± 6.5	4.9
**Post-preparative stability ** **in biological samples ** **(+4 °C, 24 h)**	Plasma	LQC	95.4 ± 3.2	7.8
		HQC	89.5 ± 3.7	6.1
	Brain	LQC	99.8 ± 2.4	10.2
		HQC	100.8 ± 1.9	6.5
	Olfactory bulb	LQC	97.7 ± 4.1	1.5
		HQC	97.5 ± 0.8	11.9
**Freeze–thaw stability ** **in biological samples**	Plasma	LQC	94.8 ± 2.4	12.8
		HQC	88.7 ± 1.8	13.6
	Brain	LQC	110.1 ± 6.4	1.0
		HQC	97.6 ± 4.7	0.6
	Olfactory bulb	LQC	99.4 ± 6.4	5.9
		HQC	101.4 ± 1.1	4.8
**Long-term stability ** **in biological samples ** **(−20 °C, 22 days)**	Plasma	LQC	100.8 ± 9.4	1.5
		HQC	87.5 ± 0.4	0.5
	Brain	LQC	96.8 ± 1.3	5.8
		HQC	96.4 ± 5.4	4.9
	Olfactory bulb	LQC	95.7 ± 8.1	1.9
		HQC	93.4 ± 0.9	2.5
